# Dormant Flower Buds Actively Accumulate Starch over Winter in Sweet Cherry

**DOI:** 10.3389/fpls.2018.00171

**Published:** 2018-02-15

**Authors:** Erica Fadón, María Herrero, Javier Rodrigo

**Affiliations:** ^1^Unidad de Hortofruticultura, Centro de Investigación y Tecnología Agroalimentaria de Aragón (CITA), Instituto Agroalimentario de Aragón (IA2), Universidad de Zaragoza, Zaragoza, Spain; ^2^Department of Pomology, Estación Experimental de Aula Dei (CSIC), Zaragoza, Spain

**Keywords:** chilling accumulation, flower buds, flower primordia, starch, sweet cherry, endodormancy

## Abstract

Temperate woody perennials survive to low temperatures in winter entering a dormant stage. Dormancy is not just a survival strategy, since chilling accumulation is required for proper flowering and arbitrates species adaptation to different latitudes. In spite of the fact that chilling requirements have been known for two centuries, the biological basis behind remain elusive. Since chilling accumulation is required for the normal growth of flower buds, it is tempting to hypothesize that something might be going on at this particular stage during winter dormancy. Here, we characterized flower bud development in relation to dormancy, quantifying changes in starch in the flower primordia in two sweet cherry cultivars over a cold and a mild year. Results show that, along the winter, flower buds remain at the same phenological stage with flower primordia at the very same developmental stage. But, surprisingly, important variation in the starch content of the ovary primordia cells occurs. Starch accumulated following the same pattern than chilling accumulation and reaching a maximum at chilling fulfillment. This starch subsequently vanished during ecodormancy concomitantly with ovary development before budbreak. These results showed that, along the apparent inactivity during endodormancy, flower primordia were physiologically active accumulating starch, providing a biological basis to understand chilling requirements.

## Introduction

Woody perennials, in temperate and boreal regions, cease growth and enter into a dormant stage to survive to low winter temperatures (Considine and Considine, 2016). But dormancy is not just a survival strategy, since cold winter temperatures are required for proper flowering (Rohde and Bhalerao, 2007; Kurokura et al., 2013), and these chilling requirements are the main drawback to cultivate temperate trees in warmer latitudes (Campoy et al., 2011).

Since this paradoxical chilling requirement was early noticed (Knight, 1801), it has been subsequently widely confirmed in a number of species and circumstances for temperate fruit tree production. Moreover, chilling requirements are specific for each cultivar and are an important criterion at the time of cultivar selection for a particular area (Atkinson et al., 2013; Jones et al., 2015). While this empirical knowledge is widely used in fruit production around the world, the question on why chilling is required has remained an unresolved mystery over these two centuries, since the biological basis of winter bud dormancy remain elusive (Fadón and Rodrigo, 2017). In an attempt to understand dormancy, terminology has been developed. Thus, in temperate fruit trees, the regulation by physiological factors inside the affected structure is referred as endodormancy, and the regulation by environmental factors is referred as ecodormancy (Lang et al., 1987), but other terms have been coined around dormancy in different species and the subject has been recently comprehensively reviewed (Considine and Considine, 2016).

The need to understand the unpredictable effects of winter rising temperatures (Fu et al., 2015) has oriented efforts to understand the genetic regulation of bud dormancy in horticulture and forestry (Abbott et al., 2015), but genetic studies are scattered and difficult to contextualize because a biological frame is missing hampering progress (Jones et al., 2015; Fadón et al., 2015a; Fadón and Rodrigo, 2017). While flower development is a highly conserved process, seasonality affects flower development in the Rosaceae (Kurokura et al., 2013). *Prunus* genus, which contains all the stone fruits, blooms in late winter or early spring, but flower buds develop over several months, with flower initiation occurring at the end of the previous summer (Kurokura et al., 2013). Recent work in sweet cherry (*Prunus avium*) shows that flower development is halted in the winter, but most interesting this halting occurs at a precise developmental stage of flower primordium (Fadón, 2015). Since chilling accumulation is required for proper flowering, it is tempting to hypothesize that at this particular developmental stage some changes should be taking place during winter dormancy that are required for subsequent proper flowering and fruiting.

On a whole plant basis, in temperate woody perennials, carbohydrates are synthesized and mainly stored in wood and roots during the growing season, up to the end of the summer, to decline first slowly through the dormant season, and then rapidly when growth is resumed (Loescher et al., 1990). In *Prunus* sp, as in other histerant species, blooming occurs before leaf emergence, and anthesis and fertilization take place with very little or complete absence of foliar area (Keller and Loescher, 1989). Thus, the reproductive process relies on stored reserves within the flower. Starch is already present upon flower opening (Rodrigo et al., 2000; Alcaraz et al., 2010), playing a clear part in reproductive success in temperate fruit tree species (Pimienta and Polito, 1982; Rodrigo and Herrero, 1998). While we know that starch has to accumulate somehow before flower opening, we do not know when and how this starch accumulates within the developing flower. Since the flower bud remains dormant for several months in sweet cherry, an exam on the starch content in the flower primordia in this period may throw light on this point. In this work, we characterized flower bud development in relation to dormancy, quantifying changes in starch in the flower primordia in two sweet cherry cultivars over a cold and a mild year. The determination of breaking of endodormancy for both cultivars allowed us estimating their chilling requirements and provided a basis to study changes in the flower primordium in relation to the dormancy status.

## Materials and Methods

### Plant Material and Sample Collection

Three trees of sweet cherry (*Prunus avium*) cultivars Burlat and Bing were selected for the experiment from a collection held at the Centro de Investigación y Tecnología Agroalimentaria (CITA), Zaragoza, Spain, at 41°44′30″ N, 0°47′00″ W and 220 m above sea level on a fine-loamy and calcareous soil. The 21-year-old trees were grafted on Santa Lucia 64 rootstock (*Prunus mahaleb*).

### Determination of Breaking of Endodormancy

For the estimation of breaking of endodormancy, shoots were sampled from the autumn, November 30, until the onset of budbreak, at the end of February or early March. Each week, three shoots (15–30 cm length and 5 mm diameter) containing at least 10 flower buds, were randomly collected from each cultivar. Dormancy status of shoots from each collecting day was determined by the evaluation of the response of the flower buds after a week in the growth chamber (Fadón and Rodrigo, 2017). To have a reference point, 10 flower buds were also randomly picked in the field and individually weighted. Shoots were placed on water soaked florist foam and maintained in a growth chamber at 22 ± 1°C with a 12-h light photoperiod. After a week in the growth chamber, 10 flower buds were randomly picked from the shoots and individually weighted. The date of breaking of endodormancy was established when the weight of the flower buds after a week in the growth chamber increased at least 30% over the buds weighted directly from the field (Brown and Kotob, 1957).

### Estimation of Chilling and Heat Requirements

Temperature was recorded hourly at a meteorological station located in the experimental orchard at CITA. Winter chilling accumulation was calculated according the “Utah Model,” which better fits in cooler areas of temperate zones (Dennis, 2003). This model proposes the sum of “chill-units” (CUs), which establish a different chilling contribution for different temperature ranges (Richardson et al., 1974). CUs were calculated from the beginning of the autumn, since in our conditions no cold temperatures were recorded before this date, until the date of breaking of endodormancy, establishing the specific CUs required for each cultivar.

Heat requirements was estimated using the Growing Degree Hours (GDH) model developed by Richardson et al. (1974) by the calculation of the GDH accumulated from the breaking of endodormancy until bloom. GDH were calculated by subtracting 4.5°C from each hourly temperature between a threshold of 6 and 25°C. All temperatures above 25°C were assumed equal to 25°C.

### Phenology and Flower Development Characterization

Phenology and flower development characterization were performed over 2 years. Seven flower buds per cultivar were randomly collected every 2 weeks from the end of the summer, in September, to bud burst in February–March. Flower buds were sampled on spurs, where are located most of the flower buds in sweet cherry trees (Herrero et al., 2017).

Phenological characterization was carried out through field observation of flower buds, which were classified following the BBCH code for sweet cherry (Fadón et al., 2015b). To characterize flower development, two flower buds per collecting date were used. Inside each flower bud, one out of the three flower primordia hosted were dissected and examined under a binocular microscope and photographed with a DC-300 digital camera (Leica Microsystems, Cambridge, United Kingdom). Flower development was classified by a 10 stages flower developmental scale developed for sweet cherry (Fadón, 2015).

### Microscope Preparations

The other five flower buds sampled per cultivar and collecting date were fixed, after removing the external scales, in ethanol (95%)/acetic acid 3:1 (v/v) over 24 h, and then transferred to ethanol (75%) at 4°C for conservation (Williams et al., 1999).

For histochemical examination, two individual flower primordia per date were embedded in Historesin (Leica, Heidelberg, Germany), sectioned at 5 μm in a Leica EM UC6 ultramicrotome with a glass knife and then place onto distilled water on a glass slide previously coated with 1% gelatine. A set of Historesin-sections were stained with periodic acid Schiff’s reagent (PAS) for insoluble carbohydrates, and counterstained with toluidine Blue for general histological observations (Feder and O′Brien, 1968). Another set of Historesin-sections were stained for DNA wint 0.05M DAPI (Williams et al., 1999), and 0.0035% Calcofluor for cellulose (Hughes and McCully, 1975) at a ratio of 1:6; over 5 min. All the preparations were observed under a bright field Leica DM2500 microscope (Leica Microsystems, Cambridge, United Kingdom). Fluorescent preparations were observed with UV epifluorescence source, using a 340–380 bandpass and a 425 longpass filter. Micrographs were taken with a DFC-310 digital camera linked to the Leica Application Suites Version 4.2.0 (Leica Microsystems, Cambridge, United Kingdom).

For starch quantification, a set of three flower buds were dehydrated in a tertiary butyl alcohol series (70, 85, 95, and 100%, v/v), embedded in paraffin wax, sectioned at 10 μm in a Jung 2045 rotatory microtome (Leica Microsystems, Cambridge, United Kingdom) and placed onto glass slides previously coated with Haupt’s adhesive. Prior to staining, the sections were rehydrated [three washes of 5 min in Histoclear II (CellPath, Hemel, United Kingdom), one in Histoclar II:ethanol (1:1, v/v) for 5 min, and one in an ethanol series (100, 70, and 40%, v/v) for 2 min]. The samples were then stained using the potassium iodide-iodine reaction (I_2_KI) for 5 min and mounted with Histomount (National Diagnostics, Atlanta, United States) (Rodrigo and Herrero, 1998). Ovary starch was qualitatively evaluated by observations under a bright field Leica DM250 microscope (Leica Microsystems, Cambridge, United Kingdom).

### Starch Quantification and Determination of Ovary Size by Image Analysis

**FIGURE 1:**
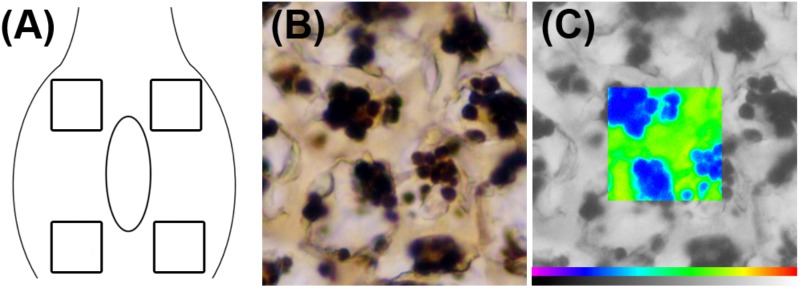
Starch quantification in sweet cherry ovary primordium. **(A)** Diagram of the ovary primordium, showing the regions in which starch content was measured. **(B)** Image of an I_2_KI-stained sample for starch. **(C)** Pseudocolor image corresponding to starch (in blue) on the black and white original image.

Starch in the flower primordium is highly compartmentalized in the different tissues, being a very small amount available for carbohydrate extraction or other analytical methods (Chow and Landhausser, 2004). As an alternative, the starch content of the ovary could be evaluated with the help of an image analysis system fitted to a microscope. After microscopic observation of I_2_KI-stained preparations, the images were photographed using a DC-300 digital camera (Leica Microsystems, Cambridge, United Kingdom) connected to the microscope. Each image was processed using a Quantiment Q550 Image Analysis System (Leica Microsystems, Cambridge, United Kingdom). Starch quantification was made by measuring the optical density of black and white images using the method described by Rodrigo et al. (1997) with modifications. In each section, four different measures were made (**Figure 1A**). The dimension of the frame used to quantify the starch in each area was 1337 μm^2^, which represents the general layout of starch in the sweet cherry ovaries. Starch granules were clearly distinguished from the background after I_2_KI staining (**Figure 1B**) and they were recognized by the image analyzer. The system identifies an individual color as the combination of three colors: red, green, and blue. Color thresholds for starch were set by increasing the detection levels until the size of the detected binary image exactly reflected the stained starch granules observed both under the microscope and in the digitalized image. These levels were stored and used for all preparations. Thus, a binary image of starch was obtained in each observation and used as a superimposed mask on the black and white original image (**Figure 1C**). The optical density of every pixel under the mask was measured, and the sum of all the values of the pixels was considered as the value of the starch content in the measured frame. The starch content was determined in six ovaries per collecting date.

To relate possible variations in the nutritive status of the ovary primordium with morphological changes, the relationship between starch content and ovary size was studied. For this purpose, the ovary diameter and the number of cell layers in each ovary was quantified in the same preparations used to measure starch content. Thus, the preparations were washed in distilled water, stained with 0.07% calcofluor white for cellulose (Hughes and McCully, 1975) and observed under a DM 250 microscope (Leica Microsystems, Cambridge, United Kingdom) with UV epifluorescence using a BP-405 exciter filter and an Y455 barrier filter. Cells were counted along the cross-section of the ovary.

### Statistical Analyses

Statistical analyses were performed using SPSS 12.0 statistical software (SPSS Inc., Chicago, IL, United States). One-way ANOVA was performed to analyze the starch content and the number of ovary cell layers. When ANOVA generated a significant *F*-value mean separations were determined by Duncan’s multiple-range test. Correlations between flower starch content and the number of chilling hours accumulated until chilling fulfillment were calculated for the two cultivars over 2 years. Once chilling was fulfilled, flower starch content was correlated with GDH accumulation. Correlations were analyzed by Pearson’s correlation coefficients at the 0.01 significance level.

## Results

### Changes along Ovary Development

**FIGURE 2:**
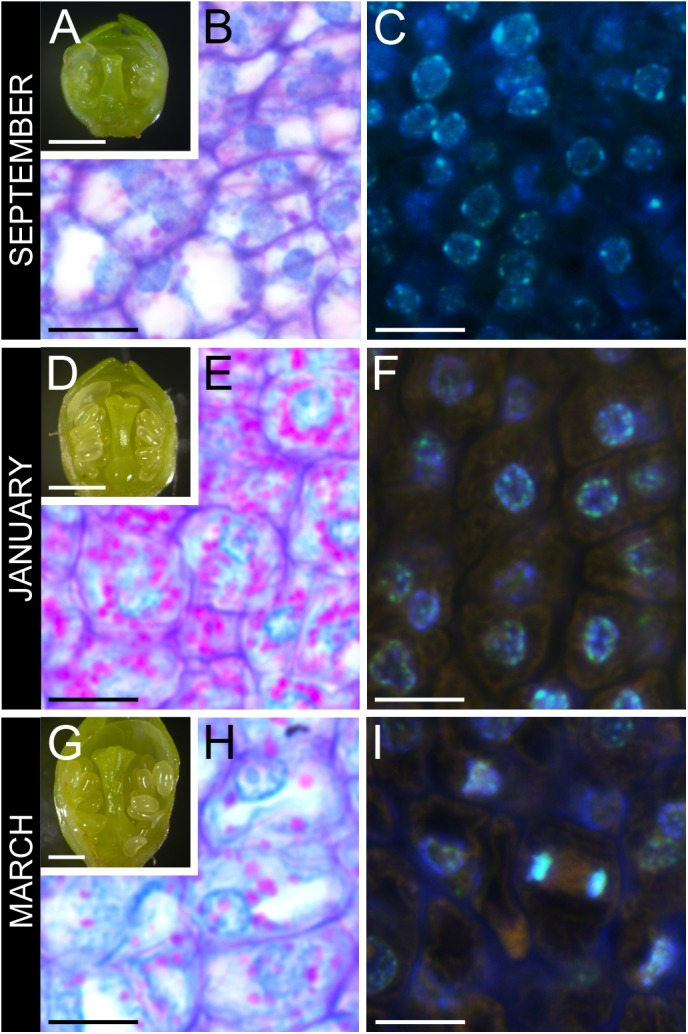
Ovary tissue before, during, and after winter dormancy in sweet cherry flower buds. **(A)** Flower primordium before dormancy at developmental stage 3, **(B)** with ovary cells showing little starch, and **(C)** active nuclei with a spongy appearance. **(D)** Flower primordium during winter dormancy at developmental stage 4, **(E)** with cells full of starch, **(F)** showing condensed nuclei. **(G)** Flower primordium after winter dormancy at developmental stage 5, **(H)** with vacuolated cells with reduced starch, and **(I)** mitotic activity. Bars **(A,D,G)** 500 μm, **(B,C,E,F,H,I)** 20 μm.

Flower primordia started developing at the end of the summer showing incipient verticiles: sepals, petals, anthers, and pistil (**Figure 2A**). Ovary cells had slight amounts of starch (**Figure 2B**), and nuclei had a spongy appearance, reflecting euchromatin (**Figure 2C**). Flower buds arrested development at late-October and enter dormancy. No anatomical changes could be detected all throughout endodormancy (**Figure 2D**). Still, in the ovary, starch accumulated within a dense cytoplasm (**Figure 2E**), and nuclei had a more condensed appearance, reflecting heterochromatin (**Figure 2F**). Following endodormancy, flower buds increased in size, and papilla started to develop in the stigma (**Figure 2G**). Starch in the ovary decreased, as the ovary cells vacuolized, increasing in size (**Figure 2H**), and divided (**Figure 2I**).

**FIGURE 3:**
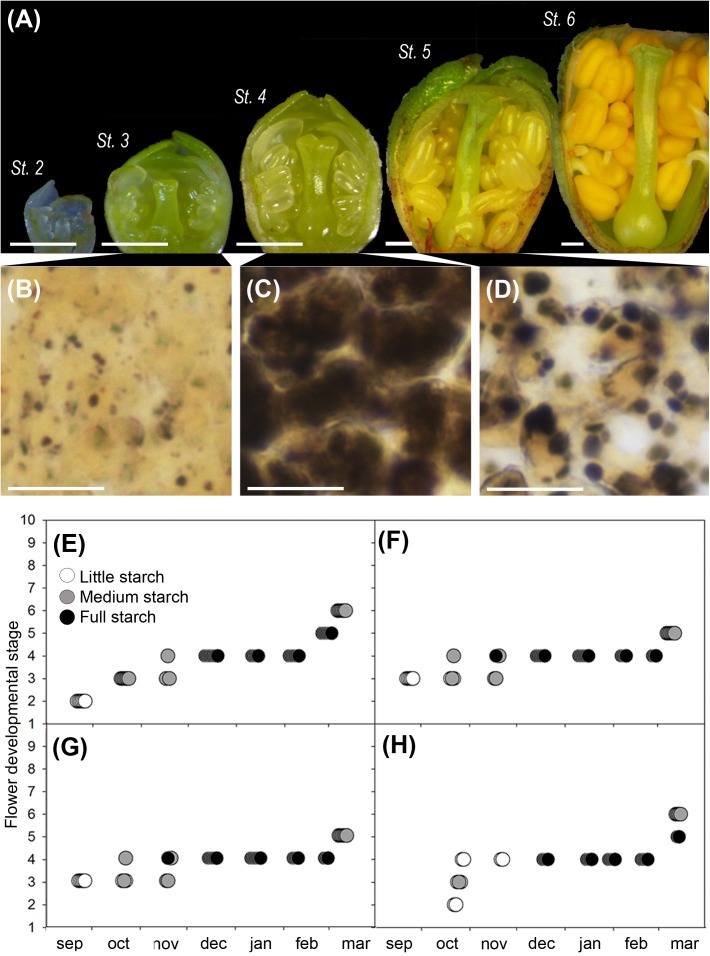
Flower development and qualitative ovary starch evaluation in sweet cherry flower buds. **(A)** Flower developmental stages (Fadón, 2015): stage 2 (St. 2), early differentiation of the flower whorls; stage 3 (St. 3), the pistil form the suture line; stage 4 (St. 4), incipient ovary, style, and stigma are apparent; stage 5 (St. 5), stigmatic papilla start developing in the spring; stage 6 (St. 6), anthers turn into bright yellow. **(B)** Histological appearance of ovary cells: with little starch before dormancy (St. 3), **(C)** full starch content during dormancy (St. 4), and **(D)** medium starch content after dormancy (St. 5). **(E–H)** Qualitative ovary starch in relation with flower developmental stage: **(E)** ‘Bing,’ cold year. **(F)** ‘Bing,’ mild year. **(G)** ‘Burlat,’ cold year. **(H)** ‘Burlat,’ mild year. Bars **(A)** 500 μm, **(B–D)** 20 μm.

Flower buds developed along the autumn (stages 2–4) and arrested development during dormancy (stage 4), to resume growth in the spring (stages 5 and 6) (**Figure 3A**). Observation of starch in the ovary cells showed a steady accumulation along the winter months (**Figures 3B,C**) and a progressive decrease after winter (**Figure 3D**). Ovary cells were devoid of starch at the early developmental stages, but starch started to accumulate at stage 3, when flower whorls had developed and clearly further built up in the dormant stage 4 (**Figures 3E–H**). Following winter dormancy, starch remained at stage 5, to decrease progressively, as flower primordia continued growth at stage 6. These observations were consistent in the two cultivars studied, over the two different years.

### Starch Accumulated during Dormancy

**FIGURE 4:**
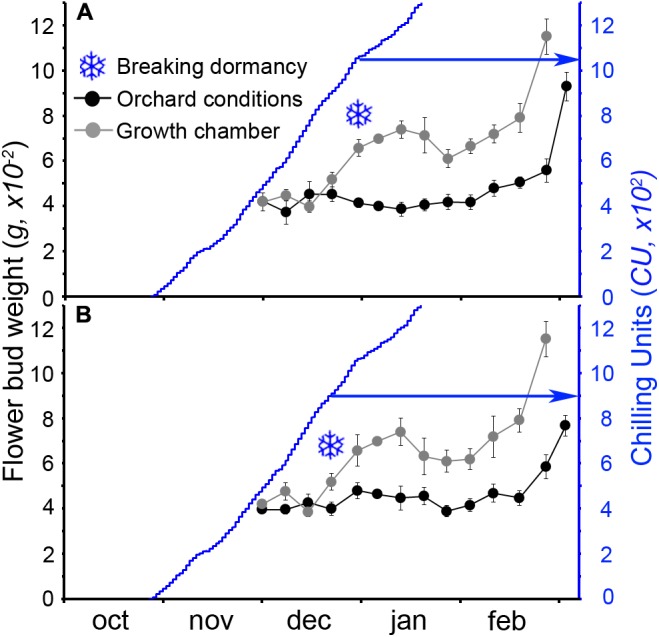
Estimation of breaking of endodormancy in two sweet cherry cultivars. Flower bud weight in orchard conditions and after a week in the growth chamber. Snowflakes: increment of at least 30%, when chilling was considered fulfilled. **(A)** Cultivar “Bing.” **(B)** Cultivar “Burlat” (n = 10).

The calculation of the number of CUs accumulated until the breaking of endodormancy showed that cultivar Burlat had lower chilling requirements (981 ± 83 CU) than Bing (1082 ± 27 CU) (**Figure 4**). In spite to the fact that the 2 years monitored had different winter temperatures, chilling accumulation followed a similar pattern, with a slow rate in the autumn, followed by a fast accumulation in the winter.

**FIGURE 5:**
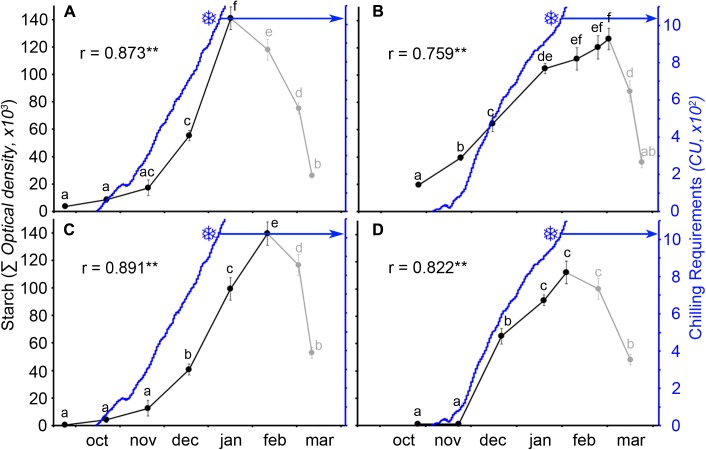
Ovary starch content (solid circles) and chilling unit accumulation (blue line) in two sweet cherry cultivars during 2 years. **(A)** ‘Bing,’ cold year. **(B)** ‘Bing,’ mild year. **(C)** ‘Burlat,’ cold year. **(D)** ‘Burlat,’ mild year. Snowflakes: dates of chilling fulfillment. *r*, Pearson’s correlation coefficients between the starch content and the chilling unit accumulation for each cultivar and year. ^∗^ P < 0.05; ^∗∗^*P* < 0.01 (*n* = 6). Different letters (a–f) indicate significant differences (*P* < 0.05) using the Duncan’s multiple-range test.

The quantitative analysis of starch in the ovary primordia showed that starch accumulated following a similar pattern in the two cultivars over the 2 years. A slow accumulation at early autumn was followed by a rapid accumulation in the following months to reach a maximum at different times for the two cultivars and years (**Figure 5**). Facing chilling accumulation to starch accumulation in the ovary, the two events followed a close pattern. This was so for both cultivars and years with a significant positive correlation between chilling and starch accumulation, until chilling fulfillment. Interestingly, differences in weather conditions were also reflected in the pattern of starch accumulation. Chilling started to accumulate about 20 days earlier in the cold (**Figures 5A,C**), than in the mild winter (**Figures 5B,D**), resulting in a 20–25 days difference between both years for the chilling fulfillment of each cultivar. Thus, in the cold year, both chilling hours and starch content accumulated earlier and quicker than in the mild year.

### Starch Vanished Following Chilling Fulfillment As Heat Accumulated

**FIGURE 6:**
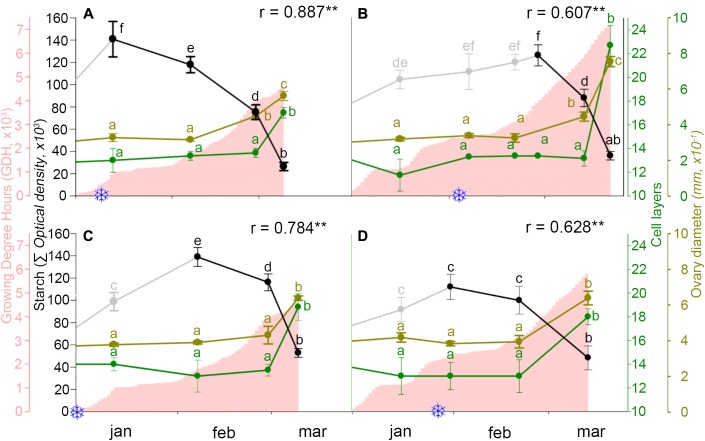
Ovary starch content (gray/black line), heat accumulation (pink area), and ovary growth as number of cell layers of the ovary wall (green line) and ovary diameter (brown line), in two sweet cherry cultivars during 2 years. **(A)** ‘Bing,’ cold year. **(B)** ‘Bing,’ mild year. **(C)** ‘Burlat,’ cold year. **(D)** ‘Burlat,’ mild year. Snowflakes: dates of chilling fulfillment. r, Pearson’s correlation coefficients between the starch content and heat accumulation for each cultivar and year. ^∗^P < 0.05; ^∗∗^P < 0.01. Different letters (a–f) indicate significant differences (*P* < 0.05) using the Duncan’s multiple-range test.

Following the breaking of endodormancy, starch did not further accumulate, but started to decrease and progressively vanished along ecodormancy in the weeks before bud burst (**Figure 5**). To evaluate whether starch decrease had a reflection on ovary growth, both were related to heat accumulation. While heat accumulation was also different over the 2 years, being lower in the cold year (**Figures 6A,C**) than in the mild year (**Figures 6B,D**), in all circumstances starch decreased following an inverse pattern with heat accumulation, with a significant negative correlation.

The number of the ovary cell layers and the ovary size followed a similar pattern. Active cell division and ovary growth occurred in the early autumn, until the flower primordia reached the dormant stage. At this point, 13–14 cell layers formed the ovary and remained during endodormancy and starch accumulation. Following early starch vanishing during ecodormancy, the number of cell layers and ovary size increased, as heat accumulated, between the end of February and mid-March, in the weeks before bud burst (**Figure 6**).

## Discussion

Results herein showed that, along the apparent inactivity during endodormancy, cherry flower primordia were physiologically active and starch accumulated in the ovary cells. Quantification of starch accumulation by image analysis fitted with the accumulation of chilling until the fulfillment of the chilling requirements. This starch subsequently vanished during ecodormancy concomitantly with ovary development before bud burst in the spring.

### A Restless Rest: Starch Accumulates along Endodormancy

Results showed that, along endodormancy, starch actively accumulated in the ovary primordia of sweet cherry flower buds. This occurred within the same flower primordium developmental stage (Fadón, 2015). In contrast with this lack of morphological changes in the flower primordia, an intense and consistent increase in starch content occurred in the ovary cells along endodormancy, supporting physiological activity in the flower primordia during this period. The transition to endodormancy has been related to changes in hormone regulation (Horvath et al., 2003), but physiological changes along endodormancy had not previously put forward (Fadón and Rodrigo, 2017). Histochemical works have showed differences in carbohydrate content in dormant buds of different woody species (Felker et al., 1983; Rinne et al., 1994; Khodorova et al., 2010). However, these differences have not been detected when analytical techniques have been used to quantify the content of carbohydrates (Bonhomme et al., 2005; Ito et al., 2012). This may have been hampered by the small size of the buds, together to the variety of the different organs and structures hosted. The approach used in this work, focusing on the ovary primordia, and combining histochemistry with a sequential quantification of starch with image analysis, showed to be a suitable method to detect changes in starch in small dormant structures.

Results showed that starch accumulated in the ovary at a particular flower developmental stage, in which all the flower whorls were differentiated. This was consistent in two different sweet cherry cultivars over 2 years. Interestingly, this particular developmental stage has been shown to be consistent for dormancy in other sweet cherry cultivars along different years (Fadón, 2015), and also in apricot (Julian, 2008). Moreover, starch has been reported in flower primordia at this developmental stage in apricot (Julian, 2008), and sour cherry (Felker et al., 1983). The question remains on whether starch also accumulates in the ovary at that particular developmental stage in other species. Further work is required to elucidate this point, but interestingly in *Arabidopsis thaliana*, this flower stage has the longest duration (Smyth et al., 1990) and starch turnover occurred during ovule and early embryo development (Hedhly et al., 2016). This opens a way to further elucidate whether starch accumulation could be conserved, even in species with a continuous flower development. Still, in temperate trees as cherries, this accumulation of starch in the winter reflects a good synchronization with the seasons and provides a key adaptive advantage.

### The Pattern of Starch Accumulation Closely Fits with Cold Accumulation

A high and significant correlation was recorded between accumulation of starch and chilling hours, and this pattern varied in the cold and mild year in a consistent way. Whether starch accumulation is triggered by cold, or is a reflection of arrested development and reduced metabolism, being the flower a clear sink for carbohydrate accumulation (Loescher et al., 1990), needs further work. Still these results may result in a more comprehensive understanding of chilling requirements, acknowledged for the last two centuries (Knight, 1801), but so far puzzling and unexplained. While low temperatures are no required to stop growth and enter endodormancy (Lang et al., 1987; Heide, 2008; Kurokura et al., 2013), the beginning of starch accumulation here reported occurred concomitantly with the beginning of chilling accumulation. This also had a reflection on the end of endodormancy that varied between years, and also between cultivars, in accordance with their different chilling requirements. Still, in all circumstances, the maximum peak of starch accumulation corresponded with the accomplishment of chilling requirements.

After chilling fulfillment, bud remained in an ecodormancy status during several weeks when starch progressively vanished and was inversely correlated with heat accumulation. Following starch decrease, the ovary cells, which had been quiescent over months, showed a decondensed nuclear chromatin, cell division, and vacuolization, as indicators of cellular activity (Horvath et al., 2003), that was reflected in the subsequent ovary growth.

### Biological Implication of Starch Accumulation during Endodormancy

The question remains on what it is the physiological significance of starch accumulation in the flower primordia during endodormancy. Changes in starch in relation to cold acclimation have been described in mature leaves, in which conversely rapid acclimation to cold is associated with starch hydrolysis, resulting in sugar increase (Kaplan et al., 2007) that reduces free water in the vacuoles (Schulze et al., 2012), preventing freezing. These opposite results could be related to the cellular differences between these two systems. In leaves, highly vacuolated mature cells are present, while in ovary primordia cells are meristematic with a dense cytoplasm lacking vacuoles, reduced water availability, and little expression of aquaporins (Yooyongwech et al., 2008).

While it remains to be elucidated if starch has a role at the time of accumulation, it is clear that this starch accumulated during winter endodormancy plays a clear part in subsequent flower development and in the reproductive process. This is in line with the fact that all along flower biology, cycles of starch accumulation and subsequent depletion do occur in different flower structures before key reproductive events, as pollen tube growth through the style (Herrero and Dickinson, 1979; Rodrigo et al., 2009), ovule development (Arbeloa and Herrero, 1991; Rodrigo and Herrero, 1998; Rodrigo et al., 2000; Hedhly et al., 2016); ovary growth (Rodrigo et al., 2000; Alcaraz et al., 2010; Hedhly et al., 2016), and fruit set (Jean and Lapointe, 2001; Ruiz et al., 2001; Iglesias et al., 2003; Lebon et al., 2004; Alcaraz et al., 2013). Results herein clearly showed that starch accumulation in the ovary primordia occurred during the apparently inactive dormant stage, concomitantly with chilling accumulation. It is tempting to put forward that lack of cold would result in failures in starch accumulation. Work in controlled temperature chambers with sour cherry twigs showed this response (Felker et al., 1983), and further work would elucidate this point. If this is so, it would provide a substrate to understand the lack of adaptation of temperate fruit tree species to warmer latitudes and failures in flowering and fruit set due to declining winter chilling (Atkinson et al., 2013).

Taken together, these results showed that in both sweet cherry cultivars analyzed herein and over both years, a restless rest occurred during endodormancy, with an intense accumulation of starch in the ovary of flower primordia, concomitantly with chilling accumulation. This provides a biological basis to understand dormancy and chilling requirements, but also may provide a frame for future studies.

## Author Contributions

EF, MH, and JR conceived, designed the study, and wrote the paper. EF performed the microscope observations and analyzed the data.

## Conflict of Interest Statement

The authors declare that the research was conducted in the absence of any commercial or financial relationships that could be construed as a potential conflict of interest.
